# Editorial: Plant-rhizobia symbiosis and nitrogen fixation in legumes

**DOI:** 10.3389/fpls.2024.1392006

**Published:** 2024-03-11

**Authors:** Senjuti Sinharoy, Chang-Fu Tian, Jesús Montiel

**Affiliations:** ^1^ Plant-Microbe Interaction, National Institute of Plant Genome Research (NIPGR) New Delhi, New Delhi, India; ^2^ State Key Laboratory of Plant Environmental Resilience, College of Biological Sciences, China Agricultural University, Beijing, China; ^3^ Center for Genomic Sciences, National Autonomous University of Mexico, Cuernavaca, Mexico

**Keywords:** legumes, symbiosis, nodule, rhizobia, nitrogen fixation

Nitrogen (N) is essential for life, but eukaryotes lack the ability to access this element, as only prokaryotic enzymes can convert N to ammonia. The Haber-Bosch process revolutionized agriculture by enabling synthetic N-fertilizer production, but its overuse and mismanagement created significant environmental challenges (Rockstrom et al., 2009; Richardson et al., 2023). Biological Nitrogen Fixation (BNF) by diazotrophic bacteria and symbiotic nitrogen fixation (SNF) by N-fixing plants offer age-old solutions to the N-problem (Adams et al., 2018).

In this context, legumes represent a valuable biological resource to migrate into a sustainable agriculture, since in N-deficient soil, they engage in symbiosis with rhizobia. In the rhizosphere, nodulation factors (NFs) secreted by rhizobia prompt mitotic activity in the root cortex cells, triggering de-differentiation and nodule formation. Concurrently, rhizobia invade root hair cells, guided by plant-derived infection threads (ITs), towards dividing plant cells. Once inside, rhizobia are endocytosed and become enclosed by plant membrane leading to the formation of ‘symbiosomes’, where they multiply and function as nitrogen-fixing entities. This Research Topic encompasses eleven articles addressing both bacterial and plant aspects essential for SNF (Kang et al., 2016; Roy et al., 2020), and the agronomical benefits of this mutualistic association.

## Legumes

It is estimated that around 25% of nodulating legumes employ an alternative rhizobial colonization process, called intercellular infection, where bacteria invade the host plant between the epidermal cells or by crack entry (Quilbe et al., 2022). Since the molecular basis of this mechanism is largely unknown, García-Soto et al. conducted a large-scale mutant screening to discover genes recruited in the intercellular symbiotic colonization of the *Agrobacterium pusense* strain IRBG74 on *Lotus japonicus* roots. The forward genetic approach was followed by sequencing the flanking regions of the mutagen to locate the potential causative genes.

Iron is crucial for various rhizobial and plant enzymes essential for BNF, including regulatory proteins like FixL and FixJ (Gilles-Gonzalez et al., 1991 *)*, nitrogen fixing enzymes NifH and NifDK (Dixon, 1998) and plant protein leghemoglobin (Wang et al., 2019; Brear et al., 2013). Iron transfer to nodules occurs through both apoplastic and symplastic routes and involves several transporters (Brear et al., 2013; Kryvoruchko et al., 2018; Tejada-Jiménez et al., 2015). MtVTL8 (Vacuolar Iron Transporter (VIT)-Like) is a major transporter responsible for delivering iron to the symbiosome in *Medicago truncatula* (Walton et al., 2020). In this Research Topic, Cai et al. highlights MtVTL8’s unconventional mechanism of iron transport across the symbiosome membrane (SM), as revealed through genetic, structural prediction, and biochemical analyses.

The SNF imposes a significant energy burden on plants due to its high photosynthetic cost. Legumes regulate nodule number using a systemic signalling called autoregulation of nodulation (AON). The SUPER NUMERARY NODULES (MtSUNN), a leucine-rich repeat receptor-like kinase in the shoot, perceives root-derived peptide signals (MtCLE12 and MtCLE13) (Kang et al., 2016; Kassaw et al., 2017; Imin et al., 2018). *MtCLE12/13* is activated in the root by the master transcription factor of nodulation, Nodule Inception (NIN) (Laffont et al., 2020). Thomas and Frugoli discuss in this Research Topic, how the MtBAM2 protein, an ortholog of the Arabidopsis BARELY ANY MERISTEM family, collaborates with SUNN, crucial for nodule meristem establishment. Additionally, Shen and Feng provide a review detailing NIN’s multifaceted role from infection to symbiosome development and AON.

Legumes also inhibit nodule formation under nitrogen sufficient condition (Kang et al., 2016) and interestingly, this effect can be partially alleviated by the organic macromolecule humic acid (HA). Zhang et al. further explored this phenomenon, by analysing the transcriptomic response of soybean nodules treated with HA under high nitrogen levels. Limited availability of phosphorus has a negative impact on nodule formation (Sulieman et al., 2013). Molecular studies using Arabidopsis have elucidated mechanisms controlling inorganic phosphate homeostasis. Under phosphate sufficiency, PHOSPHATE2 (PHO2) protein facilitate phosphate transporter degradation, limiting uptake. In phosphate deficiency, *microRNA399* (*miR399*) promotes PHO2 degradation (Aung et al., 2006; Bari et al., 2006). Huertas et al. demonstrated that MtPHO2b and MtPHO2c genes play a crucial role in regulating SNF by modulating plant phosphate homeostasis. Importantly, the complex molecular network in the legume-rhizobia symbiosis includes posttranscriptional regulation of gene expression by small RNAs (Roy et al., 2020), which are associated to ARGONAUTE proteins. Sánchez-Correa et al. explored the impact of RNAi-mediated silencing of Argonaute5 in roots and nodules of *Phaseolus vulgaris*. Additionally, they detected small RNAs bound to PvAGO5 at different stages of the nodulation process.

## Rhizobia

Rhizobial nodulation factors (NFs) and type III secretion effectors (T3SEs) are key players in NF-dependent and NF-independent nodulation processes, respectively. It is not rare for rhizobia to harbour both nodulation genes and genes encoding type III secretion system (T3SS) and T3SEs. Available evidence from the intensively studied species *Sinorhizobium fredii* supports a regulatory network for these key symbiosis genes involving transcriptional factors NodD1, SyrM, NodD2, NolR and TtsI. In this Research Topic, Navarro-Gómez et al. compared transcriptional profiles, symbiotic performance and NF composition of *S. fredii* HH103 derivatives related to these regulatory genes, and proposed an updated regulatory model. NodD1 activates the transcription of nodulation genes and TtsI that in turn upregulates T3SS/T3SE genes. On the other hand, NodD1 can also activate the transcription of *syrM*-*nodD2* and *syrM*-*nolR* modules, and NodD2 and NolR negatively regulate TtsI and NodD1 regulons. Moreover, negative regulation of *nodD2* by NolR, feedback repression of *syrM* by NodD2 and NolR, antagonistic repression between TtsI and SyrM, and autoregulation of NodD1, SyrM, NolR and TtsI were proposed. Further *in vivo* and *in vitro* protein-DNA interaction evidence can be helpful for clarifying these regulatory effects as direct and/or indirect output. The work by Navarro-Gómez et al. provides novel insight into the overlooked complexity in the regulation network of key symbiosis genes, which deserves further exploration to guide engineering elite rhizobial inoculants.

Rhizobia can live saprophytically in bulk soils, colonize rhizosphere and rhizoplane, live as host endophytes, enter intracellular symbiosis with compatible legume plants, and release from senescent nodules. In this Research Topic, Agudelo et al. reviewed related literatures on the role of direct or indirect microbial interactions that alter rhizobial fitness in rhizosphere, during nodulation and within nodules. This is a timely summary of multipartite interactions involving legumes, rhizobia and other microorganisms. Authors further highlighted several understudied issues e.g. the significance of microbial interactions within nitrogen-fixing and senescent nodules, and after nodule senescence; genetic bases and eco-evolutionary dynamics underlying microbial interactions. Further efforts in addressing these questions by recruiting a multidisciplinary strategy involving genetics, physiology, molecular biology, ecology and evolution can be helpful.

## Agricultural impact

In the last decades the benefits of SNF in agriculture has been largely documented. However, this extraordinary mutualistic association can be further exploited. In this regard, Xu et al. discovered that a mixed planting of *Medicago sativa* and *Bromus inermis* resulted in significantly higher hay yield compared to a monoculture grassland. For farmers, a key aspect to implement SNF in the field, is the prediction of the expected yield. This represents a challenging task, considering the biotic and abiotic factors that influence SNF. However, Jemo et al. conducted a multifactorial analysis using mapped soil properties and weather variables, to predict by machine-learning techniques soybean yield in fields supplemented with phosphorus and inoculated with rhizobium.

## Author contributions

SS: Conceptualization, Writing – original draft, Writing – review & editing. C-FT: Conceptualization, Writing – original draft, Writing – review & editing. JM: Conceptualization, Writing – original draft, Writing – review & editing.

## References

[B1] Adams M. A. Buchmann N. Sprent J. Buckley T. N. Turnbull T. L. (2018). Crops, nitrogen, water: are legumes friend, foe, or misunderstood ally? Trends Plant Sci. 23, 539–550. doi: 10.1016/j.tplants.2018.02.009 29559299

[B2] Aung K. Lin S. I. Wu C. C. Huang Y. T. Su C. L. Chiou T. J. (2006). pho2, a phosphate overaccumulator, is caused by a nonsense mutation in a microRNA399 target gene. Plant Physiol. 141, 1000–1011. doi: 10.1104/pp.106.078063 16679417 PMC1489903

[B3] Bari R. Datt Pant B. Stitt M. Scheible W. R. (2006). PHO2, microRNA399, and PHR1 define a phosphate-signaling pathway in plants. Plant Physiol. 141, 988–999. doi: 10.1104/pp.106.079707 16679424 PMC1489890

[B4] Brear E. M. Day D. A. Smith P. M. (2013). Iron: an essential micronutrient for the legume-rhizobium symbiosis. Front. Plant Sci. 4. doi: 10.3389/fpls.2013.00359 PMC3772312 24062758

[B5] Dixon R. (1998). The oxygen-responsive NIFL-NIFA complex: a novel two-component regulatory system controlling nitrogenase synthesis in gamma-proteobacteria. Arch. Microbiol. 169, 371–380. doi: 10.1007/s002030050585 9560416

[B6] Gilles-Gonzalez M. A. Ditta G. S. Helinski D. R. (1991). A haemoprotein with kinase activity encoded by the oxygen sensor of Rhizobium meliloti. Nature 350, 170–172. doi: 10.1038/350170a0 1848683

[B7] Imin N. Patel N. Corcilius L. Payne R. J. Djordjevic M. A. (2018). CLE peptide tri-arabinosylation and peptide domain sequence composition are essential for SUNN-dependent autoregulation of nodulation in Medicago truncatula. New Phytol. 218, 73–80. doi: 10.1111/nph.15019 29393515

[B8] Kang Y. Li M. Sinharoy S. Verdier J. (2016). A snapshot of functional genetic studies in Medicago truncatula. Front. Plant Sci. 7. doi: 10.3389/fpls.2016.01175 PMC4977297 27555857

[B9] Kassaw T. Nowak S. Schnabel E. Frugoli J. (2017). ROOT DETERMINED NODULATION1 is required for. Plant Physiol. 174, 2445–2456. doi: 10.1104/pp.17.00278 28592666 PMC5543944

[B10] Kryvoruchko I. S. I. S. Routray P. Sinharoy S. Torres-Jerez I. Tejada-Jiménez M. Finney L. A. L. A.. (2018). An iron-activated citrate transporter, MtMATE67, is required for symbiotic nitrogen fixation. Plant Physiol. 176, 2315–2329. doi: 10.1104/pp.17.01538 29284744 PMC5841734

[B11] Laffont C. Ivanovici A. Gautrat P. Brault M. Djordjevic M. A. Frugier F. (2020). The NIN transcription factor coordinates CEP and CLE signaling peptides that regulate nodulation antagonistically. Nat. Commun. 11, 3167. doi: 10.1038/s41467-020-16968-1 32576831 PMC7311451

[B12] Quilbe J. Montiel J. Arrighi J. F. Stougaard J. (2022). Molecular mechanisms of intercellular rhizobial infection: novel findings of an ancient process. Front. Plant Sci. 13. doi: 10.3389/fpls.2022.922982 PMC9260380 35812902

[B13] Richardson K. Steffen W. Lucht W. Bendtsen J. Cornell S. E. Donges J. F.. (2023). Earth beyond six of nine planetary boundaries. Sci. Adv. 9, eadh2458. doi: 10.1126/sciadv.adh2458 37703365 PMC10499318

[B14] Rockstrom J. Steffen W. Noone K. Persson A. Chapin F. S. 3rd Lambin E. F.. (2009). A safe operating space for humanity. Nature 461, 472–475. doi: 10.1038/461472a 19779433

[B15] Roy S.. (2020) 32, 15–41. NLM (Medline).

[B16] Sulieman S. Ha C. V. Schulze J. Tran L. S. (2013). Growth and nodulation of symbiotic Medicago truncatula at different levels of phosphorus availability. J. Exp. Bot. 64, 2701–2712. doi: 10.1093/jxb/ert122 23682114 PMC3697940

[B17] Tejada-Jiménez M. Castro-Rodríguez R. Kryvoruchko I. Lucas M. M. Udvardi M. Imperial J.. (2015). Medicago truncatula natural resistance-associated macrophage Protein1 is required for iron uptake by rhizobia-infected nodule cells. Plant Physiol. 168, 258–272. doi: 10.1104/pp.114.254672 25818701 PMC4424012

[B18] Walton J. H. Kontra-Kováts G. Green R. T. Domonkos Á. Horváth B. Brear E. M.. (2020). The Medicago truncatula Vacuolar iron Transporter-Like proteins VTL4 and VTL8 deliver iron to symbiotic bacteria at different stages of the infection process. New Phytol. 228, 651–666. doi: 10.1111/nph.16735 32521047 PMC7540006

[B19] Wang L. Rubio M. C. Xin X. Zhang B. Fan Q. Wang Q.. (2019). CRISPR/Cas9 knockout of leghemoglobin genes in Lotus japonicus uncovers their synergistic roles in symbiotic nitrogen fixation. New Phytol. 224, 818–832. doi: 10.1111/nph.16077 31355948

